# Neuropathic Pain and Distinct CASPR2 Autoantibody IgG Subclasses Drive Neuronal Hyperexcitability

**DOI:** 10.1212/NXI.0000000000200423

**Published:** 2025-06-25

**Authors:** Margarita Habib, Anna-Lena Wiessler, Patrik Fischer, Michele Niesner, Mareike Selcho, Ligia Abrante, Christian Werner, Annemarie Sodmann, Maximilian Koch, Abdolhossein Zare, Harald Prüss, Justina Dargvainiene, Jan Lewerenz, Robert Handreka, Peter Körtvelyessy, Dirk Reinhold, Franziska S. Thaler, Kalliopi Pitarokoili, Robert J. Kittel, Michael Briese, Michael Sendtner, Heike Rittner, Frank Leypoldt, Claudia Sommer, Robert Blum, Kathrin Doppler, Carmen Villmann

**Affiliations:** 1 Institute for Clinical Neurobiology, University Hospital Wuerzburg, Germany;; 2 Department of Neurology, University Hospital Wuerzburg, Germany;; 3 Department of Animal Physiology, Institute of Biology, Leipzig University, Germany;; 4 Institute of Clinical Chemistry, University Hospital Schleswig-Holstein Kiel/Lübeck, Germany;; 5 Department of Biotechnology and Biophysics, Biocenter, Julius-Maximilians-University Wuerzburg, Germany;; 6 Department of Neurology and Experimental Neurology, Charité Universitätsmedizin Berlin, Germany;; 7 German Center for Neurodegenerative Diseases (DZNE) Berlin, Germany;; 8 Department of Neurology, University Hospital Ulm, Germany;; 9 Department of Neurology, Medical University Lausitz Carl Thiem, Cottbus, Germany;; 10 Labor Berlin, Innovations, Berlin, Germany;; 11 Institute for Molecular and Clinical Immunology, University Magdeburg, Germany;; 12 Institute of Clinical Neuroimmunology, University Hospital, Ludwig-Maximilians-University Munich, Germany;; 13 Biomedical Center (BMC), Medical Faculty, Ludwig-Maximilians-University Munich, Martinsried, Germany;; 14 Department of Neurology, St. Josef Hospital, Ruhr-University Bochum, Germany;; 15 Center for Interdisciplinary Pain Medicine, Department of Anesthesiology, Intensive Care, Emergency and Pain Medicine, University Hospital Wuerzburg, Germany; and; 16 Department of Neurology, University Hospital Schleswig-Holstein and Kiel, Germany.

## Abstract

**Background and Objectives:**

Patients with autoantibodies (aAbs) against the contactin-associated protein-like 2 (CASPR2) suffer from a variety of clinical syndromes including neuropathic pain. CASPR2 is an adhesion protein of the neurexin family and part of the voltage-gated potassium channel complex (VGKC complex) in dorsal root ganglia (DRG) neurons. The pathologic mechanisms following the binding of CASPR2 aAbs and their association with pain are only partially understood. CASPR2 aAbs are mainly of the IgG4 subclass; however, previous studies have neglected subclass-dependent effects.

**Methods:**

We investigated 49 subclassified patient serum samples positive for CASPR2 aAbs combining superresolution lattice structural illumination microscopy (SIM^2^) and functional readouts by calcium imaging and electrophysiologic recordings on cultured DRG neurons. CASPR2-positive patient sera subclassified in IgG4 together with at least 1 other IgG subclass (IgGX) and patients with only IgG4 were further subdivided into the pain and no pain groups.

**Results:**

A decrease of CASPR2 expression along the axons after exposure to CASPR2 aAbs was observed for all patient groups except the group without pain and IgG4. Moreover, binding of CASPR2 aAbs from patients with pain increased the distance between CASPR2 and associated potassium channels along DRG axons determined by SIM^2^ microscopy. CASPR2 aAbs of patients with pain significantly increased overall neuronal excitability of cultured DRG neurons as measured by calcium imaging. Patch-clamp recordings revealed significantly decreased current amplitudes of voltage-gated potassium (Kv) channels after incubation with all 4 CASPR2 aAb subclassifications with the most prominent effect of serum samples harboring IgG4 aAbs only. Replacement of patient aAbs by healthy control serum rescued Kv channel function to normal levels suggesting that the affected potassium channel function is due to structural blockage and disrupted interactions within the VGKC complex. The last might also be rescued on novel protein synthesis and membrane trafficking of CASPR2.

**Discussion:**

IgG4 aAbs seem to be the major modifier of potassium channel function. The DRG hyperexcitability is primarily due to impaired Kv channel conductance as a consequence of CASPR2 aAb binding. However, additional unidentified signal pathways contribute to this process in patients with neuropathic pain.

## Introduction

Autoantibodies (aAbs) against contactin-associated protein-like 2 (CASPR2) are associated with various clinical syndromes including limbic encephalitis, Morvan syndrome, peripheral nerve hyperexcitability syndrome, ataxia, pain, and sleep disorders.^1-3^ CASPR2 aAb-mediated diseases often exhibit clinical relapses (25%) mostly observed if immunotherapy is discontinued.^4,5^

A significant number of patients positive for CASPR2 aAbs suffer from neuropathic pain, and in some patients, this is the only symptom present.^6,7^ In our patient cohort (total of 115 patients), pain was a frequent symptom in 36% of all patients, often severe and/or even the major symptom. Besides pain severity, 2 main phenotypes were identified: (1) primarily distal-symmetric burning pain and (2) widespread pain with myalgia and cramps.^8^ However, the mechanism by which CASPR2 aAbs drive neuropathic pain is poorly understood.

As adhesion protein of the neurexin family, CASPR2 is organizing the voltage-gated potassium channel (VGKC) complex in the CNS and at juxtaparanodal sites through contactin-2 in the peripheral nervous system.^9,10^ The VGKC complex is formed by an interaction of CASPR2 to the intracellular protein 4.1b which is linked to the postsynaptic density protein 95 (PSD-95), which in turn interacts with potassium channels (Kv1.1/Kv1.2 subtype).^11-14^ The proper clustering of Kv channels is an essential factor to keep the neuronal electrical properties enabling signal transmission,^13,15^ although a direct mechanism by which CASPR2 organizes Kv channel clusters is not known.

It has been suggested that on binding of CASPR2 aAbs, the associated potassium channels of the Kv subtype are altered in their somatic membrane expression which causes hyperexcitability of neurons and thus mediates neuropathic pain.^15^ Conversely, a significant increase in Kv1.2 expression on the presence of CASPR2 aAbs has been observed in transfected HEK-293 cells and hippocampal neurons.^13^

The CASPR2 aAbs are mainly of the IgG4 subclass.^5,10,16^ Although IgG1-3 are able to crosslink proteins and induce subsequent protein internalization, IgG4 undergoes Fab-arm exchanges which renders the immunoglobulin unable to crosslink proteins.^17^ Moreover, IgG4 is incapable of activating the complement system.

The architecture of CASPR2 consists of a large extracellular domain with 8 structural domains including 4 laminin G (L1-L4) domains, 2 epidermal growth factor (EGF)-like domains, a discoidin domain, and a fibrinogen-like domain; 1 transmembrane domain; and a short intracellular C-terminal domain.^18^ The primary epitope for CASPR2 aAbs has been determined to be located in the discoidin domain.^19,20^

Dorsal root ganglia (DRG) contain sensory neurons that are active mediators in the development of neuropathic pain.^21^ They express the proteins of the VGKC complex.^22^ CASPR2 aAbs bind to DRG neurons resulting in decreased expression and function of the associated potassium channels, thereby generating hyperexcitability. The affected potassium channels have been suggested to harbor the Kv1.1 subunit determined by blocking with dendrotoxin.^15^ However, subsets of DRG neurons differ in their Kv channel (Kv1-12) expression.^23^ To discriminate Kv channel compositions, dendrotoxins and conotoxin κM-RIIIJ are used because they are potent in vitro blockers for Kv1 channels.^24,25^ In this study, we investigated the functional mechanisms of anti-CASPR2 aAbs from patients with and without pain on nociceptive neurons and the role of IgG subclasses.

## Methods

### Patients

Sera of 17 patients with anti-CASPR2 aAbs were included in the present molecular study. Those sera were selected according to clear IgG subclassification and corresponding pain phenotype and availability of sufficient patient serum for the analysis. Patients (total 115; 102 with clinical data; 49 sera with subclass analysis with 40 sera subclassified and published in a recent study^8^) were prospectively recruited at the University Hospital Würzburg, Department of Neurology, after having given written and oral informed consent or were recruited through the German Network for Research on Autoimmune Encephalitis (GENERATE). Clinical and demographic data and aAb titer from the 17 patient sera investigated in this study were taken from the patients' records or the GENERATE registry and are summarized in Table 1. Sera of 4 controls without any neurologic symptoms or pain conditions were also included.

**Table 1:** Clinical Phenotype of Patients Tested Positive for CASPR2 Autoantibodies

Patient number	Clinical phenotype	Pain widespread	Distal burning pain	Autoantibodies	IgG subclass	Patient subclassification	Serum titer	CSF titer
P1	Epileptic seizures, dysautonomia, myoclonus	n/a	n/a	CASPR2	IgG4	No pain/IgG4	1:10,000	1:1,000
P48	Epileptic seizures, cognitive deficits, hallucinations	n/a	n/a	CASPR2	IgG4	No pain/IgG4	1:32,000	1:128
P50	Cognitive deficits, myoclonus, epileptic seizures	n/a	n/a	CASPR2	IgG4	No pain/IgG4	1:3,200	1:128
P51	Singultus	n/a	n/a	CASPR2	IgG4	No pain/IgG4	1:6,400	1:400
P9^a^	Epileptic seizures, dystonia	n/a	n/a	CASPR2	IgG4 = IgG3 = IgG2	No pain/IgG4 + IgG1-3	1:500,000	1:4,000
P34	Mnestic deficits, affective lability	n/a	n/a	CASPR2	IgG4 > IgG2	No pain/IgG4 + IgG1-3	1:1,000	1:10
P40	Epileptic seizures, cognitive deficits	n/a	n/a	CASPR2	IgG4 > IgG2 > IgG1	No pain/IgG4 + IgG1-3	1:100,000	1:100
P45	Mnestic deficits, epileptic seizures	n/a	n/a	CASPR2	IgG4 > IgG2 > IgG3	No pain/IgG4 + IgG1-3	1:750,000	n/a
P47	Mild cognitive impairment	n/a	n/a	CASPR2	IgG4 > IgG1 = IgG2	No pain/IgG4 + IgG1-3	1:3,200	1:320
P7	Neuromyotonia, pain		Distal burning pain	CASPR2	IgG4	Pain/IgG4	1:1,000	1:10
P19	Sleep disorder, depression, gait ataxia		Distal burning pain	CASPR2	IgG4	Pain/IgG4	1:6,400	1:800
P38	Myalgia, hallucinations, mnestic deficits	Widespread pain: generalized myalgia		CASPR2	IgG4	Pain/IgG4	1:100	n/a
P13^a^	Myoclonus, mnestic deficits, distal burning pain and back pain		Burning and tingling pain of the legs, back pain radiating to the legs and feet	CASPR2	IgG4 > IgG2	Pain/IgG4 + IgG1-3	1:1,000	1:10,000
P32	Mnestic deficits, dysautonomia, gait ataxia, epileptic seizures		Cramps and myalgia of the legs	CASPR2	IgG4 > IgG2 = IgG3	Pain/IgG4 + IgG1-3	1:1,000	1:100
P33	Gait ataxia, pain, peripheral neuropathy		Burning pain of the lower legs and feet	CASPR2	IgG4>IgG2	Pain/IgG4 + IgG1-3	1:1,000	1:100
P46	Mnestic deficits, neuropathic pain		Distal burning pain	CASPR2	IgG4 > IgG2 = IgG3	Pain/IgG4 + IgG1-3	1:3,200	1:1,000
P35	Severe lower back pain, radiating ventrally and to the legs		Pain of the extremities	CASPR2	IgG1	Pain/IgG1	1:320	n/a

Abbreviations: CASPR2 = Contactin-associated protein 2; IgG = immunoglobulin G.

a No Ca^2+^ imaging performed.

### Ethical Statement

Experiments using the material from patients of the University Hospital Würzburg have been approved by the Ethics Committee of the Medical Faculty, University of Würzburg, Germany (101/20). Patients from external institutes approved the usage of their sera through informed consent within the German Network for Research on Autoimmune Encephalitis and the local ethics committees. Experiments with animals were approved by the local veterinary authority (Veterinäramt der Stadt Würzburg, Germany) and the Ethics Committee of Animal Experiments, i.e., Regierung von Unterfranken, Würzburg, Germany (licence no. FBVVL 568/200–324/13).

### Preparation of DRG Neurons

Adult DRG neurons were isolated from 12 to 16 weeks old CD-1 (Charles River Sulzfeld, Germany, strain 022), collected in phosphate-buffered saline (PBS), and detached with Liberase TH (5401135001) and Liberase TM (5401119001, Roche, Basel, Switzerland) with EDTA for 30 and 10 minutes at 37°C. After centrifugation (600 × g) and trituration, they were cultured on poly-l-lysine coated coverslips and maintained for 2 days in Dulbeccos's Modified Eagle Medium/F12 (1:1) with GlutaMAX (31331-028, Gibco, NY, US) supplemented with 10% fetal calf serum and 1% penicillin/streptomycin (15140-122, Gibco, NY, US), at 37°C and 5% CO_2_.

### Microfluidic Chambers

Microfluidic chambers (MFCs) (IND150, Xona Microfluidics, Temecula, US) were placed on coverslips followed by coating with poly-l-lysine (P2636, Sigma-Aldrich, Burlington, MA, US) for 24–48 hours and laminin-111 (23017-015, Thermo Fisher Scientific, Waltham, MA, US) for 3 hours. DRGs were prepared from C57Bl/6 (Jackson Laboratory, Bar Harbor, ME, US) mice at embryonic day 13 (E13) in Hanks balanced salt solution, and dissociated in trypsin (LS003707, Worthington Biochemical, Lakewood, NJ, US) for 30 minutes at 37°C. The ganglia were then triturated and preplated for 90 minutes. The cells in the supernatant were collected and centrifuged at 400 × g for 8 minutes. Cells were resuspended in neurobasal medium with 1% GlutaMAX, 2% B27 supplement, and 2% horse serum and seeded in the MFC. The somatic compartment contained from day 1 in culture: 10 ng/mL nerve growth factor (NGF) (N-100, Alomone Labs, Jerusalem, Israel), 5 ng/mL brain-derived neurotrophic factor (BDNF) and 5 ng/mL ciliary neurotrophic factor (CNTF) (both prepared by M. Sendtner, Institute for Clinical Neurobiology, University of Würzburg), and 5 ng/mL recombinant human glial cell line-derived neurotrophic factor (PeproTech, Cranbury, NJ, US). The axonal compartment contained 40 ng/mL NGF, 5 ng/mL BDNF, and 5 ng/mL CNTF. To both sides, 1 µM 5-flurodesoxyuridine (FDU) (10124860, Thermo Scientific Chemicals, Waltham, MA, US) was added. On DIV3, the medium was exchanged by medium without FDU.

### Immunocytochemistry

For live staining, HEK-293 cells 72 hours posttransfection (eMethods) or adult DRG neurons at DIV3 were incubated with anti-CASPR2 antibodies (AF5145, 1:250, R&D Systems, Minneapolis, MN, US) and/or human patient serum (1:50–1:250) in medium for 1 hour at 4°C. Cells were fixed for 30 minutes at 4°C in 4% paraformaldehyde/4% sucrose in PBS and blocked with 10% bovine serum albumin in PBS. Permeabilization was performed with 0.1% Triton-X100 while blocking. For costaining of Kv1.1 or Kv1.2, the primary antibodies (ab65790, 1:100, Abcam; 75-008, Cambridge, United Kingdom; and 1:200, NeuroMab, Davis, CA, US) were used. The secondary antibodies (713-545-147, 1:500, anti-sheep-Alexa-Fluor-488; 109-165-003, 1:500, anti-human-Cy3; 111-165-003, 1:500, anti-rabbit Cy3; 115-165-003, 1:500, anti-mouse-Cy3, all from Jackson ImmunoResearch, Ely, United Kingdom; ab99772, 1:100, anti-IgG1-fluorescein (FITC), Abcam; 9070-30, 1:100, anti-IgG2-AF488, Southern Biotech, Birmingham, AL, US; F4641, 1:100, anti-IgG3-FITC, Sigma-Aldrich, Darmstadt, Germany; and ab99815, 1:100, anti-IgG4 FITC, Abcam) were used for 1 hour at room temperature (22°C) Cells were then incubated for 5 minutes at room temperature with 4′,6-diamidino-2-phenylindol (DAPI) diluted 1:5,000 in PBS, washed and mounted in Mowiol.

### Superresolution Microscopy

Lattice structured illumination microscopy (SIM^2^) was performed using a Zeiss ELYRA 7 (Carl Zeiss Microscopy GmbH, Jena, Germany) equipped with a Plan-Apochromat 63x/1.40 oil immersion objective and HR diode 488 nm, HR DPSS 561 nm, and HR diode 642 nm lasers. Z-stacks of images were captured. The software ZEN 3.0 SR FP2 (Carl Zeiss Microscopy GmbH, Jena, Germany) was used for image processing by a two-step reconstruction algorithm where order combination, denoising, and median filtering followed by subsequent iterative deconvolution were performed. Channel alignment through affine transformations generated from z-stacks of embedded TetraspeckTM beads (Z7279, 1:1,000, Thermo Fisher Scientific, MA, US) was used for correction of chromatic aberration.

### Calcium Imaging

DRG neurons were subjected for 2 hours with patient sera or healthy control on DIV2. DRGs were treated with 5 µM Oregon Green BAPTA 1-AM (Life Technologies, Carlsbad, CA, US) for 15 minutes at 37°C. During measurements, cells were constantly perfused with HEPES-buffered artificial CSF (in mM: 4.5 KCl, 2.5 NaH_2_PO_4_, 1 MgCl_2_, 2 CaCl_2_, 120 NaCl, 10 HEPES, 25 glucose, and pH 7.4 adjusted with NaOH) using a peristaltic pump at 37°C. A BX51WI upright microscope (Olympus, Hamburg, Germany) equipped with a 20x water-immersion objective UMPLanFL numerical aperture 0.5 and a pE-4000 fluorescence illumination system (CoolLED, Andover, United Kingdom) was used for imaging at 10 Hz with a Rolera XR Mono fast 1394 CCD (Qimaging, Surrey, Canada) camera. For each condition, 5 videos per culture with 3,000 frames (300 s) were captured with the software Streampix 4.0 (Norpix, Montreal, Canada) at a rate of 10 frames per second and a binning of 2.

To define regions of interests (ROIs), the Fiji plugin “StarDist” was used with a threshold of 0.7 to identify DRGs using star-convex shapes.^26^ The software Bio7 and the Neuron Activity Tool^27^ were used to analyze the fluorescent intensity in arbitrary unit within ROIs calculating calcium activity peaks/events. Parameters used for analysis were “signal-to-noise” of 2, “average threshold” of 1, “general activity tendency” turned off, “include variance” turned to 30, and “minimum activity counts” of 2. With the number of counted total activities, the spontaneous calcium activity per minute per neuron was calculated.

### Monoclonal CASPR2 Autoantibodies

Cloning strategy, vectors, and expression/purification methods as well as the analysis of CSF cells from patients with anti-CASPR2 encephalitis were analyzed using single-cell RNA sequencing as previously described.^28^ Finally, antibody concentration was measured using a Qubit protein assay (Invitrogen Q33211). Antigen specificity was confirmed through cell-based assays in HEK-293T cell expression full-length human CASPR2 as previously described.

### Electrophysiologic Recordings

DRG neurons were subjected for 2 hours or 24 hours with patient sera or healthy control on DIV2. Whole-cell patch clamp recordings from adult DRG neurons at DIV2 measured the maximal current amplitudes (I_max_) with an EPC-10 HEKA amplifier. Cells were held at −70 mV. A voltage step protocol (-80 mV, +40 mV) with 10 mV increments and 2.5 seconds delay was applied. The extracellular solution consisted of (mM): 135 NaCl, 5 KCl, 2 MgCl_2_, 2 CaCl_2_, 5 glucose, 10 HEPES, and pH 7.3 adjusted with NaOH. The intracellular solution consisted of (mM): 140 KCl, 2 MgCl_2_, 1 CaCl_2_, 2.5 EGTA, 10 HEPES, and pH 7.3 adjusted with KOH. Recording pipettes had a resistance of 3–6 MΩ. Cells with a capacitance between 15 and 35 pF were used. Toxins (conotoxin κM-RIIIJ and α-dendrotoxin, 100 nM) were applied through OctaFlow II system (ALA Scientific Instruments, NY, US). The currents were normalized to the healthy/disease control, setting the min average value to 0% and the max average value to 100%.

### Experimental Design and Statistical Analysis

DRG neurons were incubated with patient sera pools (1:250) containing CASPR2 aAbs (titer 1:5,000 for both IgGX (pain/no pain) groups, 1:2,000 for IgG4/pain, and 1:1,000 for IgG4/no pain) for 2 hours or 24 hours before recording. If CASPR2 aAbs were replaced by healthy control serum, the control was also used in a 1:250 dilution. All experiments were performed in triplicates if not stated otherwise and done blinded for the researcher.

Electrophysiologic recordings were analyzed using RStudio, R version 4.2.2. CASPR2 and Kv1.2 expression analysis was performed by Fiji,^29^ using plugins NeuronJ^30^ and SynapCountJ.^31^ SIM^2^ images were analyzed by Imaris Software 10.2 using the Spots function, calculating the shortest distance from CASPR2 spots to Kv1.2 spots. A threshold of 0.3 µm was applied for colocalization. Statistics and plotting were performed using GraphPad Prism, version 10.1.2.

Data are represented as mean ± standard error of the mean. Normality of the data was reviewed by the Shapiro-Wilk normality test (α = 0.05). Statistical significance was calculated using a 1-way or 2-way ANOVA*.* All numbers of experiments (*N*), cells, and *p* values are given in eTables 1–6. The 0-hypothesis was rejected at a level of *p* < 0.05.

### Data Availability

Data that support the findings of this study are available from the corresponding author, on reasonable request.

## Results

### Classification of Patients According to the IgG Subclass

Our 49 patient samples (40 patient sera were subclassified to their IgG class of CASPR2 aAbs previously^8^) were screened for the predominant IgG subclass and subdivided into 4 different groups according to their pain phenotype and IgG subclass distribution: patients with pain/IgG4 only or IgG4+ additional IgG1-3 (IgGX), patients without pain/IgG4 only or IgGX. 17 selected patient serum samples were used in this study to further evaluate the CASPR2 aAb pathophysiology (Table 1).

**Figure 1:**
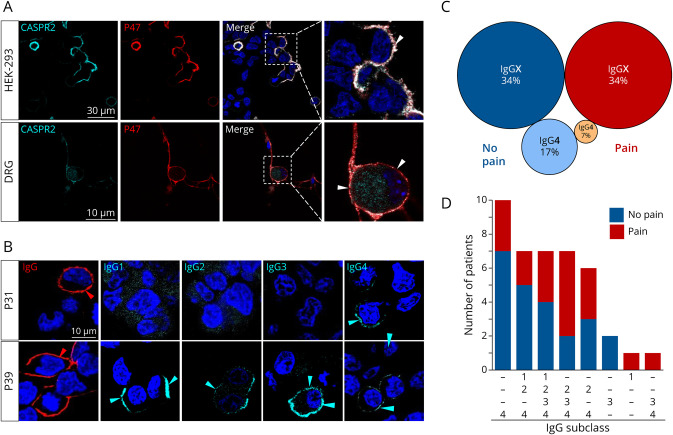
CASPR2 aAbs in Patient Serum Show Different IgG Subclass Compositions (A) Exemplary immunocytochemical stainings of CASPR2 (cyan) and patient serum (red) binding to membrane of CASPR2-transfected HEK-293 cells and adult DRG neurons. (B) Stainings for different IgG subclasses (cyan) in 2 exemplary total IgG positive (red) patient sera (P31, P39). (C) Distribution of IgG subclasses (IgG4 only or IgG4 plus additional IgG = IgGX) and pain phenotype with pain—red and orange circles, no pain—light and dark blue circles with circle size related to the number of positive patient sera. (D) Distribution of IgG subclass combinations in patients with pain (red) and without pain (blue). CASPR2 = Contactin-associated protein 2; DRG = dorsal root ganglia; IgG = immunoglobulin G.

CASPR2 aAb positive sera were first confirmed in cell-based assays and on adult mouse DRG neurons in costainings with a commercial anti-CASPR2 antibody (Figure 1A). Representative stainings of total IgG or IgG subclasses of selected patient sera show a serum positive for IgG4 only (P31) and a serum positive for all IgG subclasses (P39) (Figure 1B). Almost all patients were IgG4 positive validating IgG4 as the main subclass as previously described.^5,10^ The other 3 IgG subclasses appeared also quite prominent in the order IgG2 > IgG3 > IgG1. IgG4 was often detected together with IgG2 (about 65% of all patients), while the presence of IgG4 together with either IgG3 or IgG1 was less prominent (35%–37%). Pain and no pain were similarly distributed within the patient cohort (41%–51%, Figures 1, C and D). Pain and no pain patients with IgG4 only were less representative (7 and 17%) than patients with CASPR2 aAbs with IgG4 and at least 1 additional IgG (34% each; Figures 1, C and D). Two patients had only IgG3, 1 only IgG1, and for some other patients, either no clinical information on the pain phenotype was available or the serum amount was too little for IgG subclass determination (8%, data not shown).

### CASPR2 Expression Is Unaltered in DRGs on Long-term Presence of Anti-CASPR2 aAbs

CASPR2 belongs to the VGKC complex which is functionally expressed in DRG neurons. DRGs represent active mediators in the development of neuropathic pain to transmit pain signals from the peripheral nervous system to the CNS.^21,32^ Within the VGKC complex, CASPR2 is associated with potassium channels of the Kv subtype through intercellular proteins 4.1b and PSD-95. Colocalization of CASPR2 with Kv1.1 or Kv1.2 was verified in transfected HEK-293 cells, adult and embryonic (E13) DRG neurons at DIV2 (eFigure 1).

The pathophysiology of other aAbs often includes changes in the expression pattern of the targeted protein.^33,34^ Patients harbor CASPR2 aAbs, however, for days or even weeks before a clinical phenotype manifests. To mimic disease, CASPR2 expression was analyzed in DRG neurons incubated for 1 day, 2 days, and 4 days with patient sera. To model plasma exchange, we exchanged the serum after 2 days with a healthy control serum for 2 days (2R for the rescue group) (eFigure 2A). From immunofluorescence images, CASPR2 expression was estimated using 10 patient sera and a healthy control serum individually. Group analysis according to the pain phenotype independent of which IgG subclass revealed a significant decrease in CASPR2 expression between day 1 and day 4 for the no pain group (*p* = 0.0147) but not in the pain group (*p* = 0.9536) or for different IgG subclasses (IgG4 *p* = 0.1413; IgGX *p* = 0.4255; eFigure 2, eTable 1).

An analysis of CASPR2 density considering patient serum samples from both no pain groups (IgG4 and IgGX) separately exhibited a nonsignificant lower CASPR2 density on days 2 and 4 compared with day 1. The pain group with IgG4 revealed a slight increase in CASPR2 density along the axons but also nonsignificant (eFigure 2). In summary, the CASPR2 expression alterations in DRG neurons supplemented with CASPR2 aAbs were minor.

In a comparative approach, we used an in vivo setting to quantify nocifensive behavior elicited by mechanical *von Frey* filament stimulation in *Drosophila* larvae (eMethods).^35,36^ Whereas knockdown of the voltage-gated potassium channel *shaker* (*Drosophila* Kv1 homolog) through nociceptor-specific RNAi gave a phenotype-resembling hyperalgesia, *neurexin-IV* (*nrx-IV, Drosophila* CASPR2 homolog) knockdown in fact slightly decreased nocifensive behavior (eFigure 3). These data emphasize the evolutionarily conserved role of voltage-gated potassium channels in nociceptors and indicate that the potassium channel subunits of the VGKC complex represent excellent candidates for the nociceptive association of CASPR2 aAbs.

### The Structural Organization of the VGKC Complex Is Altered in the Presence of Anti-CASPR2 aAbs From Patients With Pain

**Figure 2:**
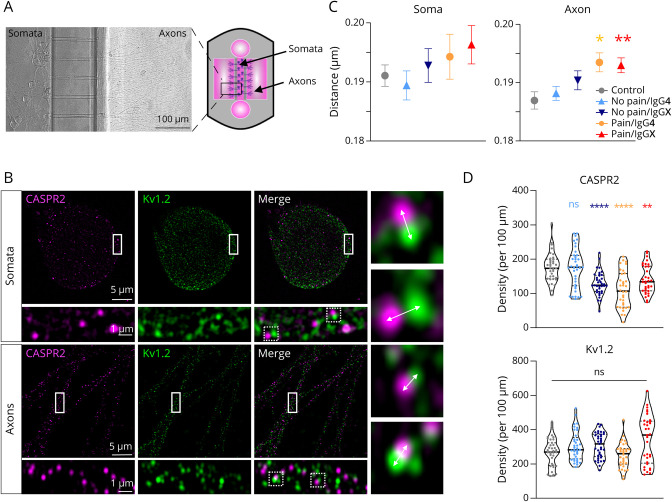
Expression of CASPR2 After Long-Term Exposure to CASPR2 aAbs and Structural Integrity of the VGKC Complex (A) Microfluidic chamber (MFC) with embryonic DRGs to separate somatic and axonal compartments and investigate VGKC complex organization after CASPR2 aAb incubation in separated compartments. (B) Exemplary pictures using lattice SIM^2^ microscopy of soma and axons from DRGs in MFCs treated with healthy control serum. Arrows mark the distance between CASPR2 (magenta) and Kv1.2 (green). (C) Quantification of distance between CASPR2 and Kv1.2 for somata (left) and axons (right) after CASPR2 aAb incubation for 2 hours. Data are shown as mean ± SEM. Somatic and axonal complexes: n = 325–1,465 and n = 1872–3,603, respectively. (D) CASPR2 and Kv1.2 density per 100 µm axon (CASPR2 n = 34–37; Kv1.2 n = 33–37). Data are shown as violin plots with individual values, median = bold line, quartiles = dotted lines. Levels of significance: ns: not significant, *p < 0.05, **p < 0.01, ****p < 0.0001. CASPR2 = Contactin-associated protein 2; DRG = dorsal root ganglia; VGKC complex = voltage-gated potassium channel complex.

Possible structural rearrangements of CASPR2 and Kv potassium channel subunit proteins of the VGKC complex were investigated with high-resolution SIM^2^ imaging. DRG neurons grown in MFCs improved resolution by clear discrimination between somatic and axonal localization (Figures 2, A and B). Somatic and axonal compartments were incubated separately with 4 different patient serum pools depending on pain phenotype and IgG subclass (Table 1) for 2 hours. Afterward, CASPR2 and Kv1.2 were stained and analyzed for their distances (Figure 2C). Although no significant distance changes were obtained for the somatic compartment, a significant increase in the distances between CASPR2 and Kv1.2 on axons was seen for both pain groups independent of the IgG composition compared with healthy control (Figure 2C, eTable 2). Hence, we observed changes in the organization of the VGKC complex on incubation with CASPR2 aAbs from patients with pain. Moreover, the CASPR2 density along the axons was significantly decreased for both pain groups independent of the IgG class and the no pain, IgGX group (Figure 2D) after presence of aAbs for 2 hours. These data differ from the long-term presence of CASPR2 aAb over several days where only minor expression changes of CASPR2 have been observed (eFigure 2). The expression analysis argues that novel CASPR2 synthesis is counteracting of CASPR2 internalization during the long-term presence of CASPR2 aAbs. Here, after short-term presence of aAbs, CASPR2 density was significantly decreased, while the cluster density of the associated Kv1.2 channels was indistinguishable in the presence and absence of CASPR2 aAbs (Figure 2D).

### Assessment of Neuronal Activity After Incubation With Anti-CASPR2 by Ca^2+^ Imaging

**Figure 3:**
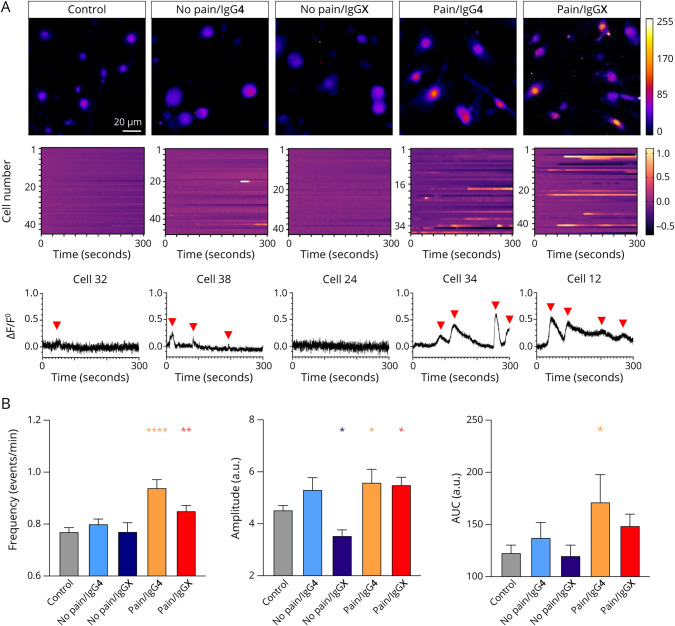
Spontaneous Calcium Activity of DRGs After Incubation With CASPR2 aAbs (A) Exemplary pictures and activity graphs of ROIs after short-term incubation with control serum or serum group with pain and IgGX. Neuronal activity from ∼40 cells is shown in a heatmap. (B) Frequency (left), amplitude (middle), and area under the curve (AUC; right) of spontaneous calcium activity events after short-term incubation with CASPR2 aAbs of different serum subclassifications. Data shown as mean ± SEM, n = 718 (control), n = 682 (no pain/IgG4), *n* = 476 (no pain/IgGX), n = 566 (pain/IgG4), and n = 727 (pain/IgGX). Levels of significance: *p < 0.05, **p < 0.01, ****p < 0.0001. aAbs = autoantibodies; CASPR2 = Contactin-associated protein 2; DRG = dorsal root ganglia.

To control for functional alterations, the excitability of adult DRG neurons was investigated in Ca^2+^imaging experiments. Two hours after CASPR2 aAb treatment, we analyzed the spontaneous activity of adult DRG neurons (Table 1, eTable 3). Fluorescent intensity traces from representative cells from all 5 conditions and additional heatmaps of ∼40 cells were analyzed (Figure 3A). After 2 hours of incubation, only the 2 groups associated with pain harboring either IgG4 or IgGX CASPR2 aAbs significantly increased the neuronal activity of DRG neurons measured by increasing calcium transient frequency, amplitude, and area under the curve (AUC) when compared with incubation with healthy control serum (frequency: pain/IgG4, *p* < 0.0001; pain/IgGX, *p* = 0.0100; amplitude: pain/IgG4, *p* = 0.0343; pain/IgGX, *p* = 0.0391; AUC: pain/IgG4, *p* = 0.0180; Figure 3B, eTable 3). Taken together, the hyperexcitability observed for adult DRG neurons after treatment with CASPR2 aAbs from patients suffering from neuropathic pain points toward a significant effect on impaired spontaneous activity at the DRG neurons.

### Effects of Anti-CASPR2 aAbs on the Function of the Associated Kv Channels

Although with small sample size (n = 2 CASPR2-positive sera), CASPR2 aAbs have been shown to decrease the function of the associated Kv channel leading to hyperexcitability of DRG neurons.^15^ Importance of IgG subtypes was not reported.

**Figure 4:**
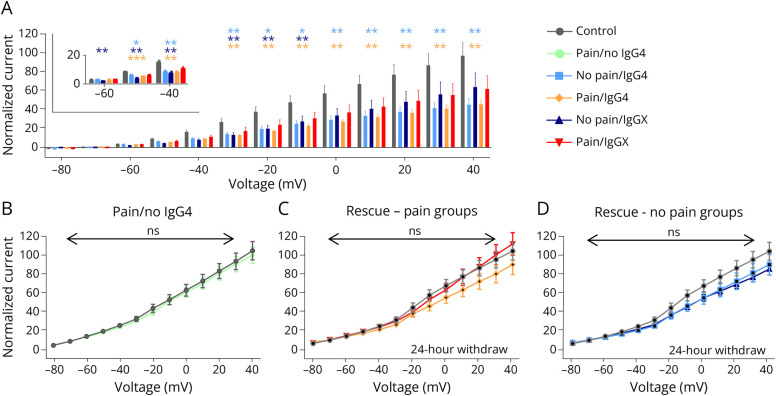
Decrease of Kv Currents on CASPR2 aAb Application (A) Bar plot of potassium channel activity at different voltage steps from −80 to +40 mV. Normalized current compared with the healthy control is shown. (B) I–V (current-voltage relation) plot measured after short-term aAb application on DRG neurons under the condition: pain/no IgG4, N = 2, n = 10. (C and D) Replacement of the CASPR2 aAbs by healthy control serum led to a rescue of affected potassium channel current (patients with pain and patients without pain), N = 2, n = 10–11. Levels of significance: *p < 0.05, **p < 0.01, ***p < 0.001. aAbs = autoantibodies; CASPR2 = Contactin-associated protein 2; DRG = dorsal root ganglia; Kv = voltage-gated potassium.

**Figure 5:**
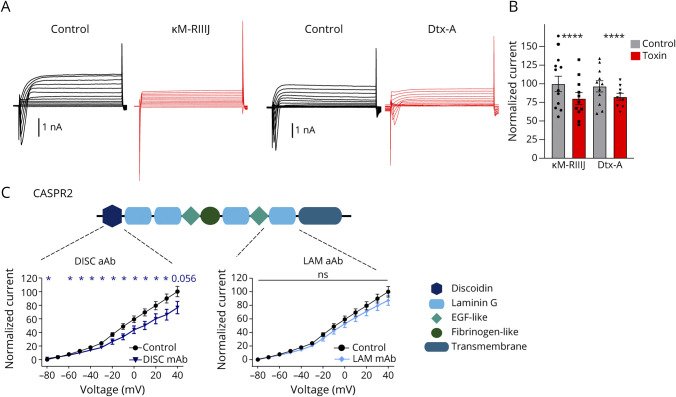
CASPR2 Monoclonal Autoantibodies Isolated From Patients Also Decrease Potassium Channel Activity in DRG Neurons (A and B) Representative potassium channel current recording and the effect of specific potassium channel subunit composition blocker α-dendrotoxin Dtx-A and κM-RIIIJ (red traces) shown in the bar plot (at +40 mV). Data shown as mean ± SEM. (C) Domain architecture of CASPR2 including the discoidin domain, fibrinogen-like domain, and laminin domains. Patch clamp recordings using a voltage step protocol from −80 to +40 mV in the presence of CASPR2 monoclonal autoantibodies against the discoidin domain (left) demonstrating significant alterations of the potassium channel activity and against the laminin domain (right) with no significant alterations. N = 3, n = 13–16. Level of significance: *p < 0.05, ****p < 0.0001. CASPR2 = Contactin-associated protein 2; DRG = dorsal root ganglia.

All 4 subgroups of CASPR2-positive patient serum samples significantly decreased the function of Kv channels (for voltage range −50 to −10, *p* < 0.05 for all groups except pain/IgGX, from 0 to 40 IgG4 groups, *p* < 0.01 (Figure 4A). Prominent impairment of potassium currents was observed for the groups with only IgG4 independent of the pain phenotype (eFigure 4). All 4 groups of patients carry IgG4 or IgG4 and an additional IgG, suggesting IgG4 as a main driver of hyperexcitable DRG neurons in the presence of CASPR2 aAbs. We next selected 1 patient with non-IgG4 but low level of IgG1 CASPR2 aAbs and did not observe alterations of the potassium channel activity after pretreatment, further providing evidence for IgG4 as a key IgG of CASPR2 aAb pathophysiology (Figure 4B). The patient serum with no IgG4 but IgG1 had a low autoantibody titer (Table 1), which may also underlie the obtained unaltered Kv channel function on binding to the associated CASPR2 target. To verify a causal effect of aAb binding, the aAbs were replaced by healthy serum exchange for 24 hours following 2 hours of the presence of the CASPR2 aAbs. This resulted in a rescue of the affected Kv channel function (Figures 4, C and D). Incubation with CASPR2 aAbs for 24 hours also caused a reduction of the Kv channel activity although less prominent (eFigure 5). Hence, our data reveal a local and direct effect of CASPR2 autoantibodies on sensory neuron excitability mediated by impaired Kv channel activity (Figure 4, eTable 4). Different current patterns were observed pointing toward a contribution of Kv1.1, Kv1.2, and possibly Kv1.1/Kv1.2 heteromers but also arguing for other Kv subtypes present. Using the specific toxin κM-RIIIJ which specifically blocks Kv1.2 heteromers, Kv1.2 heteromers were confirmed as significant portion of the recorded ion channels (*p* < 0.001). A similar portion of potassium currents could be blocked with α-dendrotoxin which blocks Kv1.1, Kv1.2, and Kv1.6 (*p* < 0.001, Figures 5A and B, eTable 5). To further evaluate that the effect was mediated by CASPR2-binding aAbs and not by aAbs targeting other neuronal components present in polyclonal patient sera, we used monoclonal anti-CASPR2 aAbs isolated from antibody-secreting cells in patients' CSF cloned to an IgG4 backbone. The IgG4 backbone was modified to prevent Fab-arm exchange and thus restore a cross-linking ability to CASPR2 IgG4 aAbs.^37,38^ The monoclonal antibodies used were known to target either the discoidin domain as a main target of CASPR2 aAbs or the laminin domain which is present 4 times in the extracellular domain of CASPR2 (Figure 5C). Although monoclonal antibodies targeting the discoidin domain reduced the Kv channel activity significantly compared with the control, this potential was not obtained for the antibodies against the laminin domains (Figure 5C). An impairment of potassium channel function has also been demonstrated for aAbs against another adhesion protein LG1 (against leucine rich repeat domain),^39^ which was used as a positive control (eFigure 6).

In summary, we document a direct effect of CASPR2 aAbs of the IgG4 subtype on the function of the associated Kv channel. Thus, IgG4 is a key driver of increased excitability of sensory DRG neurons.

## Discussion

CASPR2 aAbs have been found in patients with various neurologic diseases including neuropathic pain. We subclassified patient sera positive for CASPR2 aAbs according to their pain phenotype and IgG subclass which sheds further light on differences in pathologic mechanisms of those aAbs and their pain association.

A previous study documented an increased pain sensitivity following passive transfer of purified patient CASPR2 IgG from 2 patients into mice.^15^ Patient-derived CASPR2 IgG increased DRG excitability but only in 1 patient, while aAbs from the other patient lacked this effect. Decreased CASPR2 and Kv levels were detected at the juxtaparanodal regions from injected animals.^15^ By contrast, others have described an enhanced cluster formation of CASPR2, an increased expression of the associated potassium channel subunit Kv1.2, but an almost unaffected CASPR2 expression in transfected cells and hippocampal neurons.^13^

CASPR2 aAbs have been determined as mainly of the IgG4 subclass,^5,10,16^ hence differing from the other IgG classes by their inability to activate complement, crosslink proteins, and induce their subsequent internalization. Recently, we found that most patients with CASPR2 aAbs harbor aAbs not only of the IgG4 subclass but at least 1 other IgG subclass in addition.^8^ Thus, the additional subclass might contribute to differences in the pathologic mechanisms. Besides the importance of the IgG subclass, even domain-specific targeting effects of aAbs may play a role.

Simulating the situation in the patient before and after (plasmapheresis) treatment using a time course of 4 days and a subsequent recovery period of 2 days after aAbs withdrawal, we found a slight decrease in endogenous CASPR2 expression in treated DRG neurons for all 4 patient subclassifications (pain vs no pain and IgG4 vs IgGX). The observed decrease in CASPR2 expression after 4 days of presence of aAbs was however only significant in the no pain groups independent of the IgG subclass. These data align with unaltered or minor decrease in CASPR2 expression in primary neurons or following passive transfer of patient IgG in mice.^13,15^ After an exchange of the CASPR2 aAbs to a healthy control serum, expression levels were clearly rescued to the baseline level independent of patient subclassifications. The short-term presence of CASPR2 aAbs decreased CASPR2 density leaving the expression level of the associated potassium channel Kv1.2 unaffected. No studies, at least to our knowledge, exist for the protein half-life of CASPR2 from neuronal analysis or in vivo experiments. The half-life for CASPR2 has however been determined in transfected cells to be 3.7 hours.^40,41^ The observed slight decrease in CASPR2 expression in the presence of CASPR2 aAbs over a time frame reflecting several magnitudes of CASPR2 protein half-life is unlikely to be the main pathologic mechanism of CASPR2 aAbs.

Further structural analysis using high-resolution SIM^2^ microscopy revealed a significant increase in the distances between CASPR2 and Kv1.2, both being members of the VGKC complex, along DRG axons in the presence of CASPR2 aAbs from patients with pain suggesting that the protein complex loses its structural integrity on aAb binding. The VGKC complex structural integrity was unchanged for patients without pain. The nonpersisting structural integrity of the VGKC complex on CASPR2 aAb binding is thus similar to other aAb against adhesion proteins, e.g. LG1, where a spatial reorganization of the associated Kv channels along the axon initial segment has been found to underlie functional impairment of the affected neurons.^39^ We further concentrated on the mechanism of pain association with CASPR2 aAbs, which has been suggested to occur through hyperexcitability of DRG neurons^15^ as mediators of pain signaling to the CNS. In *Drosophila* larvae, a RNAi-mediated knockdown of the CASPR2 homolog *nrx-IV* did not elevate nocifensive behavior. By contrast, knockdown of the potassium channel subunit *shaker,* the mammalian Kv1 homolog, led to an increase in nocifensive responses. This is in line with potassium channels controlling neuronal excitability.^42,43^

Using Ca^2+^ imaging to study neuronal excitability, only patient serum samples associated with a pain phenotype significantly increased the frequency, the amplitude, and AUC of measured calcium transients. The neuronal activity pattern was almost unchanged for serum samples without pain association. If increased neuronal activity is a direct consequence of exposure to pain-associated CASPR2 aAbs and thus the functionality of the VGKC complex, similar effects would be expected from patch clamp recordings considering Kv channel conductance. Of interest, whole-cell recordings from pretreated DRG neurons with CASPR2 aAbs from all patient serum subclassifications showed significantly decreased potassium channel activity with the most prominent effect of the no pain/pain IgG4 subclasses. Replacement of aAbs by healthy control serum always rescued potassium channel activity indicating that functional alterations of recorded DRG neurons were a direct consequence of aAb binding to the VGKC complex which is present in almost all DRG subtypes.^22^ The presence of an additional IgG class slightly minimized functional DRG impairments. Similarly, CASPR2 aAbs of the IgG4 group had a more pronounced effect on the overall neuronal activity in Ca^2+^ imaging, strongly indicating that IgG4 is the main driver of DRG hyperexcitability. A decreased potassium channel activity has been previously shown but only for 1 patient sample^15^ proposing that the 2 patient samples tested may have been differed in IgG compositions of CASPR2 aAbs. Elucidating which Kv subtypes may underlie the decreased potassium channel function revealed a partial contribution of Kv1.1, Kv1.2, and Kv1.6, which are expressed in virtually all types of DRG neurons.^22,24,44^ Considering epitope-specific pathophysiologic effects of aAbs similar to recent findings for LG1 aAbs involving a spatial redistribution of Kv channels and concomitant impaired neuronal control of action potential initiation and synaptic integration,^39^ epitope-specific effects of discoidin and laminin domain targeting patient-derived CASPR2 aAbs on Kv dysfunction cannot be excluded.

**Figure 6:**
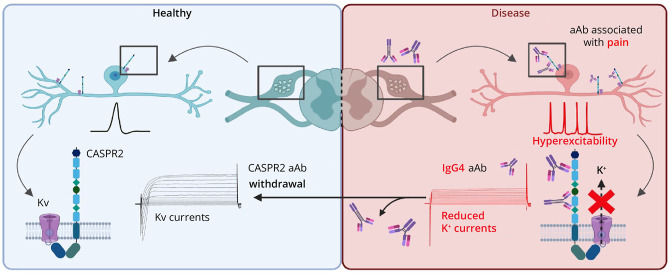
Pathophysiologic Mechanisms of CASPR2 aAbs Following Patient Subclassification According to IgG Subtype and Pain Phenotype DRG neurons are active mediators in developing neuropathic pain: healthy condition (left) and disease condition in the presence of CASPR2 autoantibodies (aAbs, right). Sensory neuron hyperexcitability is driven by pain-associated CASPR2 aAbs. This causes enhanced neuronal activity and decreased function of the associated Kv channels. Pathologic activity of sensory neurons is mainly promoted by CASPR2 aAbs of the IgG4 subtype. Created in BioRender. Villmann, C. (2025) BioRender.com/. CASPR2 = Contactin-associated protein 2; DRG = dorsal root ganglia.

Our argument that IgG4 is a key driver of decreased potassium channel activity (Figure 6) was further reinforced when using a patient serum lacking IgG4 but harboring low level of IgG1 and exhibiting no alteration of potassium channel function. However, this reinforcement likewise represents a limitation of our study. Patients with CASPR2 aAbs not representing the IgG 4 subclass are scarce. In our cohort, 1 patient with IgG1 and a low autoantibody titer and 2 patients with only IgG3 were identified, the last with no clear pain phenotype. Nevertheless, our data indicate that the pathophysiology of CASPR2 IgG4 possibly by its inability to crosslink 2 CASPR2 proteins but binding to 1 CASPR2 protein of the VGKC complex structurally hinders the activation of the associated potassium channels. The pathophysiology of CASPR2-positive patients associated with pain, however, correlates significantly with a decrease of the structural integrity of the targeted protein complex, decreased CASPR2 density, and consequently an increase of the overall neuronal excitability of cultured sensory neurons. Hence, the pathophysiology of neuropathic pain in patients with CASPR2 aAbs includes additional molecular pathways. Disorders due to neuronal hyperexcitability with and without pain have been prone to display an interplay between impaired sodium channel and potassium channel activity leading to decreased thresholds of action potential firing or changes such as prolonged repolarization phases of action potentials.^45,46^ Besides an involvement of other ion channels, specific neuronal subtypes, inflammatory pathways, or the role of immune complexes triggering secondary intracellular signal cascades further manifesting with an associated pain phenotype represent additional options. Taken together, the subclassification of CASPR2-positive patient serum samples resembles a key strategy to unravel discrepancies in the pathologic mechanisms that are the prerequisite for targeted treatment.

## Glossary

AUC: area under the curve
BDNF: brain-derived neurotrophic factor
CASPR2: contactin-associated protein-like 2
CNTF: ciliary neurotrophic factor
DRG: dorsal root ganglia
E13: embryonic day 13
FDU: flurodesoxyuridine
GENERATE: German Network for Research on Autoimmune Encephalitis
MFC: microfluidic chamber
NGF: nerve growth factor
PBS: phosphate-buffered saline
PSD-95: postsynaptic density protein 95
ROI: regions of interest
SIM^2^: structural illumination microscopy

## References

[1] Dalmau J, Geis C, Graus F. Autoantibodies to synaptic receptors and neuronal cell surface proteins in autoimmune diseases of the central nervous system. Physiol Rev. 2017;97(2):839-887. doi:10.1152/physrev.00010.2016 28298428 PMC5539405

[2] Dalmau J, Graus F. Antibody-mediated encephalitis. N Engl J Med. 2018;378(9):840-851. doi:10.1056/NEJMra1708712 29490181

[3] Gövert F, Abrante L, Becktepe J, et al. Distinct movement disorders in contactin-associated-protein-like-2 antibody-associated autoimmune encephalitis. Brain. 2023;146(2):657-667. doi:10.1093/brain/awac276 35875984

[4] Gadoth A, Pittock SJ, Dubey D, et al. Expanded phenotypes and outcomes among 256 LGI1/CASPR2-IgG-positive patients. Ann Neurol. 2017;82(1):79-92. doi:10.1002/ana.24979 28628235

[5] van Sonderen A, Ariño H, Petit-Pedrol M, et al. The clinical spectrum of Caspr2 antibody-associated disease. Neurology. 2016;87(5):521-528. doi:10.1212/WNL.0000000000002917 27371488 PMC4970662

[6] Klein CJ, Lennon VA, Aston PA, McKeon A, Pittock SJ. Chronic pain as a manifestation of potassium channel-complex autoimmunity. Neurology. 2012;79(11):1136-1144. doi:10.1212/WNL.0b013e3182698cab 22895588 PMC3525306

[7] Lancaster E, Huijbers MG, Bar V, et al. Investigations of caspr2, an autoantigen of encephalitis and neuromyotonia. Ann Neurol. 2011;69(2):303-311. doi:10.1002/ana.22297 21387375 PMC3059252

[8] Greguletz P, Plötz M, Baade-Büttner C, et al. Different pain phenotypes are associated with anti-Caspr2 autoantibodies. J Neurol. 2024;271(5):2736-2744. doi:10.1007/s00415-024-12224-4 38386048 PMC11055745

[9] Lu Z, Reddy MV, Liu J, et al. Molecular architecture of contactin-associated protein-like 2 (CNTNAP2) and its interaction with contactin 2 (CNTN2). J Biol Chem. 2016;291(46):24133-24147. doi:10.1074/jbc.M116.748236 27621318 PMC5104938

[10] Patterson KR, Dalmau J, Lancaster E. Mechanisms of Caspr2 antibodies in autoimmune encephalitis and neuromyotonia. Ann Neurol. 2018;83(1):40-51. doi:10.1002/ana.25120 29244234 PMC5876120

[11] Giannoccaro MP, Crisp SJ, Vincent A. Antibody-mediated central nervous system diseases. Brain Neurosci Adv. 2018;2:2398212818817497. doi:10.1177/2398212818817497 32166168 PMC7058213

[12] Irani SR, Vincent A. Voltage-gated potassium channel-complex autoimmunity and associated clinical syndromes. Handb Clin Neurol. 2016;133:185-197. doi:10.1016/B978-0-444-63432-0.00011-6 27112678

[13] Saint-Martin M, Pieters A, Déchelotte B, et al. Impact of anti-CASPR2 autoantibodies from patients with autoimmune encephalitis on CASPR2/TAG-1 interaction and Kv1 expression. J Autoimmun. 2019;103:102284. doi:10.1016/j.jaut.2019.05.012 31176559

[14] van Sonderen A, Petit-Pedrol M, Dalmau J, Titulaer MJ. The value of LGI1, Caspr2 and voltage-gated potassium channel antibodies in encephalitis. Nat Rev Neurol. 2017;13(5):290-301. doi:10.1038/nrneurol.2017.43 28418022

[15] Dawes JM, Weir GA, Middleton SJ, et al. Immune or genetic-mediated disruption of CASPR2 causes pain hypersensitivity due to enhanced primary afferent excitability. Neuron. 2018;97(4):806-822.e10. doi:10.1016/j.neuron.2018.01.033 29429934 PMC6011627

[16] Irani SR, Pettingill P, Kleopa KA, et al. Morvan syndrome: clinical and serological observations in 29 cases. Ann Neurol. 2012;72(2):241-255. doi:10.1002/ana.23577 22473710

[17] van der Neut Kolfschoten M, Schuurman J, Losen M, et al. Anti-inflammatory activity of human IgG4 antibodies by dynamic Fab arm exchange. Science. 2007;317(5844):1554-1557. doi:10.1126/science.1144603 17872445

[18] Poliak S, Gollan L, Martinez R, et al. Caspr2, a new member of the neurexin superfamily, is localized at the juxtaparanodes of myelinated axons and associates with K+ channels. Neuron. 1999;24(4):1037-1047. doi:10.1016/s0896-6273(00)81049-1 10624965

[19] Liang W, Zhang J, Saint-Martin M, et al. Structural mapping of hot spots within human CASPR2 discoidin domain for autoantibody recognition. J Autoimmun. 2019;96:168-177. doi:10.1016/j.jaut.2018.09.012 30337146

[20] Olsen AL, Lai Y, Dalmau J, Scherer SS, Lancaster E. Caspr2 autoantibodies target multiple epitopes. Neurol Neuroimmunol Neuroinflamm. 2015;2(4):e127. doi:10.1212/NXI.0000000000000127 26185774 PMC4496632

[21] Esposito MF, Malayil R, Hanes M, Deer T. Unique characteristics of the dorsal root ganglion as a target for neuromodulation. Pain Med. 2019;20(suppl 1):S23-S30. doi:10.1093/pm/pnz012 PMC6544557 31152179

[22] Kupari J, Usoskin D, Parisien M, et al. Single cell transcriptomics of primate sensory neurons identifies cell types associated with chronic pain. Nat Commun. 2021;12(1):1510. doi:10.1038/s41467-021-21725-z 33686078 PMC7940623

[23] Rasband MN, Park EW, Vanderah TW, Lai J, Porreca F, Trimmer JS. Distinct potassium channels on pain-sensing neurons. Proc Natl Acad Sci U S A. 2001;98(23):13373-13378. doi:10.1073/pnas.231376298 11698689 PMC60878

[24] Cordeiro S, Finol-Urdaneta RK, Kopfer D, et al. Conotoxin κM-RIIIJ, a tool targeting asymmetric heteromeric K _v_ 1 channels. Proc Natl Acad Sci U S A. 2019;116(3):1059-1064. doi:10.1073/pnas.1813161116 30593566 PMC6338859

[25] Grissmer S, Nguyen AN, Aiyar J, et al. Pharmacological characterization of five cloned voltage-gated K+ channels, types Kv1.1, 1.2, 1.3, 1.5, and 3.1, stably expressed in mammalian cell lines. Mol Pharmacol. 1994;45(6):1227-1234. doi:10.1016/s0026-895x(25)10594-4 7517498

[26] Schmidt U, Weigert M, Broaddus C, Myers G. Cell Detection with Star-Convex Polygons. Springer International Publishing; 2018:265-273.

[27] Prada J, Sasi M, Martin C, Jablonka S, Dandekar T, Blum R. An open source tool for automatic spatiotemporal assessment of calcium transients and local 'signal-close-to-noise' activity in calcium imaging data. Plos Comput Biol. 2018;14(3):e1006054. doi:10.1371/journal.pcbi.1006054 29601577 PMC5895056

[28] Esser D, Müller-Miny L, Heming M, et al. Activated alphabeta T- and reduced mucosa-associated invariant T cells in LG1- and CASPR2-encephalitis. Brain. 2025;awaf096. doi:10.1093/brain/awaf096. 40094812 PMC12404778

[29] Schindelin J, Arganda-Carreras I, Frise E, et al. Fiji: an open-source platform for biological-image analysis. Nat Methods. 2012;9(7):676-682. doi:10.1038/nmeth.2019 22743772 PMC3855844

[30] Meijering E, Jacob M, Sarria JC, Steiner P, Hirling H, Unser M. Design and validation of a tool for neurite tracing and analysis in fluorescence microscopy images. Cytometry A. 2004;58(2):167-176. doi:10.1002/cyto.a.20022 15057970

[31] Mata G, Heras J, Morales MAR, Rubio J. Synapcountj - a tool for analyzing synaptic densities in neurons. arXiv:150707800:2015.

[32] Krames ES. The dorsal root ganglion in chronic pain and as a target for neuromodulation: a review. Neuromodulation. 2015;18(1):24-32. doi:10.1111/ner.12247 25354206

[33] Carvajal-González A, Leite MI, Waters P, et al. Glycine receptor antibodies in PERM and related syndromes: characteristics, clinical features and outcomes. Brain. 2014;137(Pt 8):2178-2192. doi:10.1093/brain/awu142 24951641 PMC4107739

[34] Moscato EH, Peng X, Jain A, Parsons TD, Dalmau J, Balice-Gordon RJ. Acute mechanisms underlying antibody effects in anti-N-methyl-D-aspartate receptor encephalitis. Ann Neurol. 2014;76(1):108-119. doi:10.1002/ana.24195 24916964 PMC4296347

[35] Dannhäuser S, Lux TJ, Hu C, et al. Antinociceptive modulation by the adhesion GPCR CIRL promotes mechanosensory signal discrimination. Elife. 2020;9:e56738. doi:10.7554/eLife.56738 32996461 PMC7546736

[36] Tracey WD Jr., Wilson RI, Laurent G, Benzer S. painless, a Drosophila gene essential for nociception. Cell. 2003;113(2):261-273. doi:10.1016/s0092-8674(03)00272-1 12705873

[37] Handlogten MW, Peng L, Christian EA, et al. Prevention of Fab-arm exchange and antibody reduction via stabilization of the IgG4 hinge region. MAbs. 2020;12(1):1779974. doi:10.1080/19420862.2020.1779974 32633193 PMC7531514

[38] Rispens T, Huijbers MG. The unique properties of IgG4 and its roles in health and disease. Nat Rev Immunol. 2023;23(11):763-778. doi:10.1038/s41577-023-00871-z 37095254 PMC10123589

[39] Sell J, Rahmati V, Kempfer M, et al. Comparative effects of domain-specific human monoclonal antibodies against LGI1 on neuronal excitability. Neurol Neuroimmunol Neuroinflamm. 2023;10(3):e200096. doi:10.1212/NXI.0000000000200096 37028941 PMC10099296

[40] Falivelli G, De Jaco A, Favaloro FL, et al. Inherited genetic variants in autism-related CNTNAP2 show perturbed trafficking and ATF6 activation. Hum Mol Genet. 2012;21:4761-4773. doi:10.1093/hmg/dds320 22872700 PMC3471401

[41] Zhang Q, Sterling K, Xu L, et al. CNTNAP2 protein is degraded by the ubiquitin-proteasome system and the macroautophagy-lysosome pathway. Mol Neurobiol. 2023;60(5):2455-2469. doi:10.1007/s12035-023-03227-9 36658382

[42] Zemel BM, Ritter DM, Covarrubias M, Muqeem T. A-type K(V) channels in dorsal root ganglion neurons: diversity, function, and dysfunction. Front Mol Neurosci. 2018;11:253. doi:10.3389/fnmol.2018.00253 30127716 PMC6088260

[43] Pongs O. Regulation of excitability by potassium channels. Results Probl Cel Differ. 2008;44:145-161. doi:10.1007/400_2007_032 17579818

[44] Giacobassi MJ, Leavitt LS, Raghuraman S, et al. An integrative approach to the facile functional classification of dorsal root ganglion neuronal subclasses. Proc Natl Acad Sci U S A. 2020;117(10):5494-5501. doi:10.1073/pnas.1911382117 32079727 PMC7071849

[45] Higerd-Rusli GP, Alsaloum M, Tyagi S, et al. Depolarizing Na(V) and hyperpolarizing K(V) channels are Co-trafficked in sensory neurons. J Neurosci official J Soc Neurosci. 2022;42(24):4794-4811. doi:10.1523/JNEUROSCI.0058-22.2022 PMC9188389 35589395

[46] Higerd-Rusli GP, Tyagi S, Baker CA, et al. Inflammation differentially controls transport of depolarizing Nav versus hyperpolarizing Kv channels to drive rat nociceptor activity. Proc Natl Acad Sci U S A. 2023;120(11):e2215417120. doi:10.1073/pnas.2215417120 36897973 PMC10089179

